# Altering a Histone H3K4 Methylation Pathway in Glomerular Podocytes Promotes a Chronic Disease Phenotype

**DOI:** 10.1371/journal.pgen.1001142

**Published:** 2010-10-28

**Authors:** Gaelle M. Lefevre, Sanjeevkumar R. Patel, Doyeob Kim, Lino Tessarollo, Gregory R. Dressler

**Affiliations:** 1 Department of Pathology, University of Michigan, Ann Arbor, Michigan, United States of America; 2 Department of Internal Medicine, University of Michigan, Ann Arbor, Michigan, United States of America; 3 Neural Development Section, National Cancer Institute, Frederick, Maryland, United States of America; Medical Research Council Human Genetics Unit, United Kingdom

## Abstract

Methylation of specific lysine residues in core histone proteins is essential for embryonic development and can impart active and inactive epigenetic marks on chromatin domains. The ubiquitous nuclear protein PTIP is encoded by the *Paxip1* gene and is an essential component of a histone H3 lysine 4 (H3K4) methyltransferase complex conserved in metazoans. In order to determine if PTIP and its associated complexes are necessary for maintaining stable gene expression patterns in a terminally differentiated, non-dividing cell, we conditionally deleted PTIP in glomerular podocytes in mice. Renal development and function were not impaired in young mice. However, older animals progressively exhibited proteinuria and podocyte ultra structural defects similar to chronic glomerular disease. Loss of PTIP resulted in subtle changes in gene expression patterns prior to the onset of a renal disease phenotype. Chromatin immunoprecipitation showed a loss of PTIP binding and lower H3K4 methylation at the *Ntrk3* (neurotrophic tyrosine kinase receptor, type 3) locus, whose expression was significantly reduced and whose function may be essential for podocyte foot process patterning. These data demonstrate that alterations or mutations in an epigenetic regulatory pathway can alter the phenotypes of differentiated cells and lead to a chronic disease state.

## Author Summary

While all cells contain essentially the same genome, adult differentiated cells have specific patterns of gene expression for unique physiological functions. Gene expression depends on specific proteins that activate some genes and repress others so that a stable pattern of expression is maintained. During embryonic development, epigenetic modifications of the genome may compartmentalize the genome into actively expressed or repressed domains through the methylation of specific histone residues on chromatin. We studied a specific pathway of histone H3 lysine 4 methylation by deleting the co-factor PTIP in a differentiated cell type. We then asked whether this epigenetic pathway is still important for maintaining the correct pattern of gene expression. Using the podocyte cells of the glomerulus as a model system, mice that carry deletions of the PTIP protein only in these podocytes show changes in gene expression patterns over time and exhibit a slowly progressing chronic disease phenotype. Chromatin immunoprecipitation showed a loss of PTIP binding and lower H3K4 methylation at the *Ntrk3* locus, whose expression was significantly reduced. These data demonstrate the need for maintaining the correct epigenetic pattern in an aging, differentiated cell type and point to modifications in epigenetics as potential disease causing factors.

## Introduction

The process of embryonic development determines the differentiated state of all cells by establishing unique gene expression patterns, or signatures, for individual cell types that define their phenotypes. Once a differentiated state is established, it is difficult to erase that epigenetic imprint and reprogram the cell towards a different cell lineage or phenotype. Although reprogramming can be forced by nuclear transplantation [1] or by the expression of Oct4 and accessory factors [2], [3], the low efficiency of these processes speaks to the inherent stability of a differentiated cell. Gene expression patterns must be established and maintained by compartmentalizing the genome into active and inactive regions, which is thought to occur through the covalent modifications of DNA and its associated nucleosomes. Such modifications include DNA methylation of CpG islands and methylation, acetylation, and ubiquitination of histone tails, all of which are thought to determine chromatin structure and accessibility [4], [5]. This epigenetic code is thus imprinted upon the primary genetic code during embryonic development to help establish cell lineages and restrict fate.

The genetics and biochemistry of histone modifications have been well studied in a variety of model organisms and developmental contexts. Genes of the Polycomb and Trithorax families encode proteins that are required for methylation of different histone lysine residues and often correlate with gene silencing or activation, respectively [6]–[9]. Many Trithorax group proteins, such as *Drosophila* TRX and human KMT2A (MLL), are histone H3 lysine 4 (H3K4) methyltransferases (KMTs) and are essential for maintaining gene expression patterns in diverse organisms. Recently, we discovered a novel co-factor, PTIP (Pax Transactivation-domain Interacting Protein), which is encoded by the *Paxip1* gene. The PTIP protein co-purifies with the mammalian lysine methyltransferases KMT2B and KMT2C (formerly ALR and MLL3), is broadly expressed, and is essential for embryonic development [10]–[12]. At least in one case, PTIP is able to recruit the KMT2B complex to a developmental DNA binding protein in a locus specific manner [13]. Loss of PTIP function in the mouse results in gross developmental effects at gastrulation, with reduced levels of global H3K4 di- (me2) and trimethylation (me3) observed [13], [14]. In cultured mouse embryonic stem cells, PTIP is needed to maintain pluripotency, Oct4 expression, and normal levels of H3K4 trimethylation [15]. Similarly, in neuronal stem cells, differentiation is abrogated and levels of H3K4 methylation are reduced in tissue specific PTIP knockouts [13]. In mouse embryo fibroblasts, loss of PTIP blocks differentiation by inhibiting PPARγ and C/EPBα activation and H3K4 methylation at their respective promoters [16]. Similarly, the *Drosophila* homologue of PTIP is also essential for development, epigenetic control of gene expression, and global histone H3K4 methylation [17].

During cell division, patterns of histone methylation must be inherited by daughter cells such that the cellular phenotype is maintained. For repressive histone methylation marks, such as histone H3 lysine 27, the EED (Embryonic Ectodermal Development) protein is thought to bind and recruit the Polycomb Repressor Complex 2 to replicate and maintain gene silencing after mitotic cell division [18], [19]. For highly expressed genes, the KMT2A (MLL1) protein associates with promoter regions on condensed mitotic chromatin and is required to rapidly reactivate such genes after cell division [20]. These data suggest a model whereby histone methylation patterns are replicated during mitosis, but do not address the necessity for maintaining epigenetic modifications in terminally differentiated, non-dividing cells. Furthermore, changes in the expression of epigenetic regulatory genes have been reported in a variety of cancers [21] and disease states [22], but whether these are the cause or the result of disease remains to be determined.

To address the necessity of H3K4me3 in a stable non-dividing cell type, we utilized a Podocin-Cre transgenic driver to delete PTIP in the glomerular podocyte, a highly specialized and architecturally distinct cell that establishes the kidney filtration barrier. Podocytes are clinically relevant cells whose properties and expression profiles change in glomerular diseases and in older animals [23]. While the ubiquitous expression of PTIP, its role in H3K4 methylation, and its necessity in development and differentiation are all well established, whether PTIP deletion in terminally differentiated cells can induce changes in the pattern of H3K4me3 and gene expression has not been demonstrated. We show that loss of PTIP results in changes in the transcriptional profile of terminally differentiated podocyte cells, which ultimately leads to a chronic glomerular disease phenotype. Among the most affected is the neurotrophin receptor encoding gene *Ntrk3*, whose function had not been previously studied in podocytes. Our results demonstrate a maintenance function for PTIP-mediated H3K4 methylation and identify a novel role for *Ntrk3* in podocyte foot process patterning.

## Results

### Generation of a Podocyte-Specific *Paxip1* Deletion

**Figure 1:**
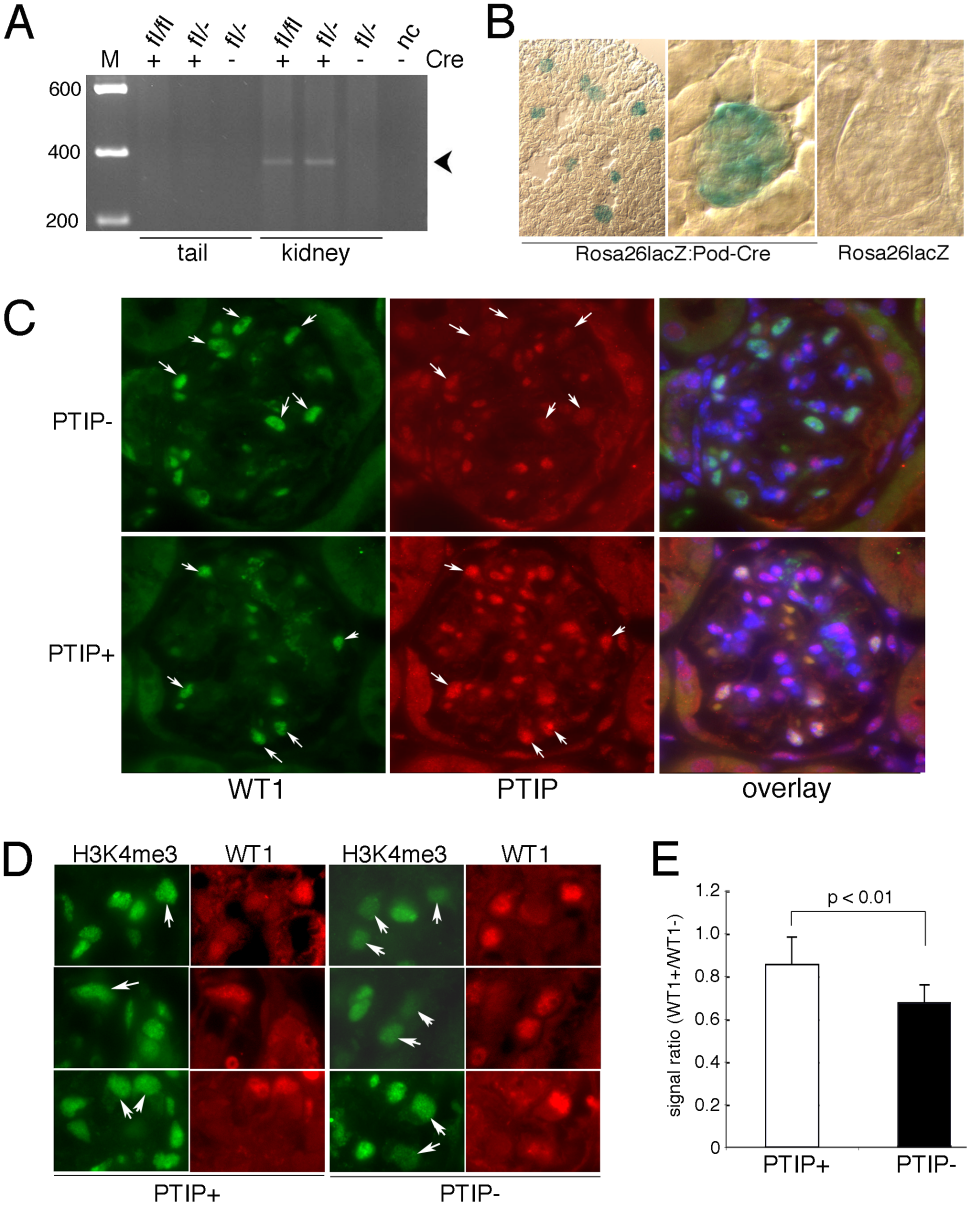
Generation of a Podocyte-Specific *Paxip1* Deletion. A) PCR genotyping with primer pairs specific for the excised, null allele indicates *Paxip1* excision only in the kidney DNA and only in mice carrying the *Cre^NPHS2^* transgene. B) Enzymatic staining for β-galactosidase activity (blue) in kidney sections from 1 month old mice with the indicated genotypes. C) Immunostaining for WT1 (green) and PTIP (red) in glomeruli at 3 months of age show reduced PTIP signals in the WT1 positive cells (arrows) of PTIP− kidneys compared to PTIP+ control littermates. The overlays were counterstained with DAPI to mark all nuclei. Thus double positives (WT1 and PTIP) are light purple whereas single positives (WT1 only) are green. D) Immunostaining for H3K4me3 and WT1 in kidneys of 3 months old PTIP+ and PTIP− mice. Note reduced intensity of podocyte cells (arrows) in PTIP− mice, when compared to other cells on the sections. E) Image analysis of immunostaining for H3K4me3 from 3 month old PTIP+ and PTIP− mice. The total signal strength was calculated by integrating over a fixed area and the data are expressed as the ratio of podocytes to mesangial and endothelial cell signals. Mean ratios from 6 podocytes and 6 other cell types were calculated from 8 independent samples for each genotype. Error bars are one standard deviation from the mean. The p value was calculated by the students t-test for 2 independent variables.

To specifically knockout PTIP protein in fully differentiated mouse podocytes, we utilized both floxed (fl) and conventional null (-) alleles of *Paxip1* and a Cre driver strain specific for glomerular podocytes. The *Paxip1* ^fl/−^:*Cre* ^NPHS2^ mice were crossed to *Paxip1* ^fl/fl^ animals to generate *Paxip1* ^fl/fl^ or *Paxip1* ^fl/−^ with or without *Cre* ^NPHS2^. The *Cre* ^NPHS2^ mice utilize the *NPHS2* promoter to express Cre recombinase only in late developing and mature podocytes [24], [25]. The resulting progenies were born in the expected Mendelian ratios and did not show any gross kidney defects during the first 4 weeks of life (data not shown). For simplicity, we will refer to the mice as either PTIP− (*Paxip1* ^fl/−^:*Cre* ^NPHS2^; *Paxip1* ^fl/fl^:*Cre* ^NPHS2^) or PTIP+ (*Paxip1* ^−/fl^, or *Paxip1* ^fl/fl^). PCR analysis indicated that recombination occurred at the *Paxip1* locus in DNAs isolated from kidneys but not in DNAs from tails (Figure 1A). Previous work established that the *Paxip1^fl^* allele produces normal levels of protein, but Cre-mediated excision of exon 1 and the promoter region results in complete absence of PTIP protein, essentially creating a null allele [13], [15]. The specificity of the Cre driver strain was confirmed by crossing *Cre* ^NPHS2^ mice to the Rosa26-LacZ reporter mice (Figure 1B). In 1 month old kidneys, lacZ expression was restricted to the glomerulus only, indicating efficient Cre mediated excision at this time. Immunostaining for PTIP and the podocyte marker WT1 also confirmed that PTIP protein levels were reduced only in the podocyte cells and not the mesangial or endothelial components of the glomerular tuft (Figure 1C). Previous work showed that a loss of PTIP function results in reduced levels of total H3K4me3 levels in embryos and cultured cells [13]–[17]. To test whether podocytes showed reduced H3K4me3, we stained kidney sections with antibodies specific for this modification (Figure 1D). Many podocytes were observed with reduced signal intensities. To quantitate this effect, images were analyzed for signal intensity by integrating a fixed area over the nuclei of both podocytes and other cell types (Figure 1E). Podocytes were co-stained with WT1 antibodies. The ratio of podocyte signal (WT1+) to other cell types (WT1−) was calculated by counting at least 6 cells of each type per glomerulus. The ratios from at least 8 glomeruli were averaged for each genotype and shown to decrease by more than 20% in PTIP− kidneys compared to PTIP+ controls (p<0.01). These data confirmed that the specific deletion of PTIP in the podocytes correlates with a reduction in H3K4me3 in this cell type.

### Development of a Chronic Glomerular Disease Phenotype

**Figure 2:**
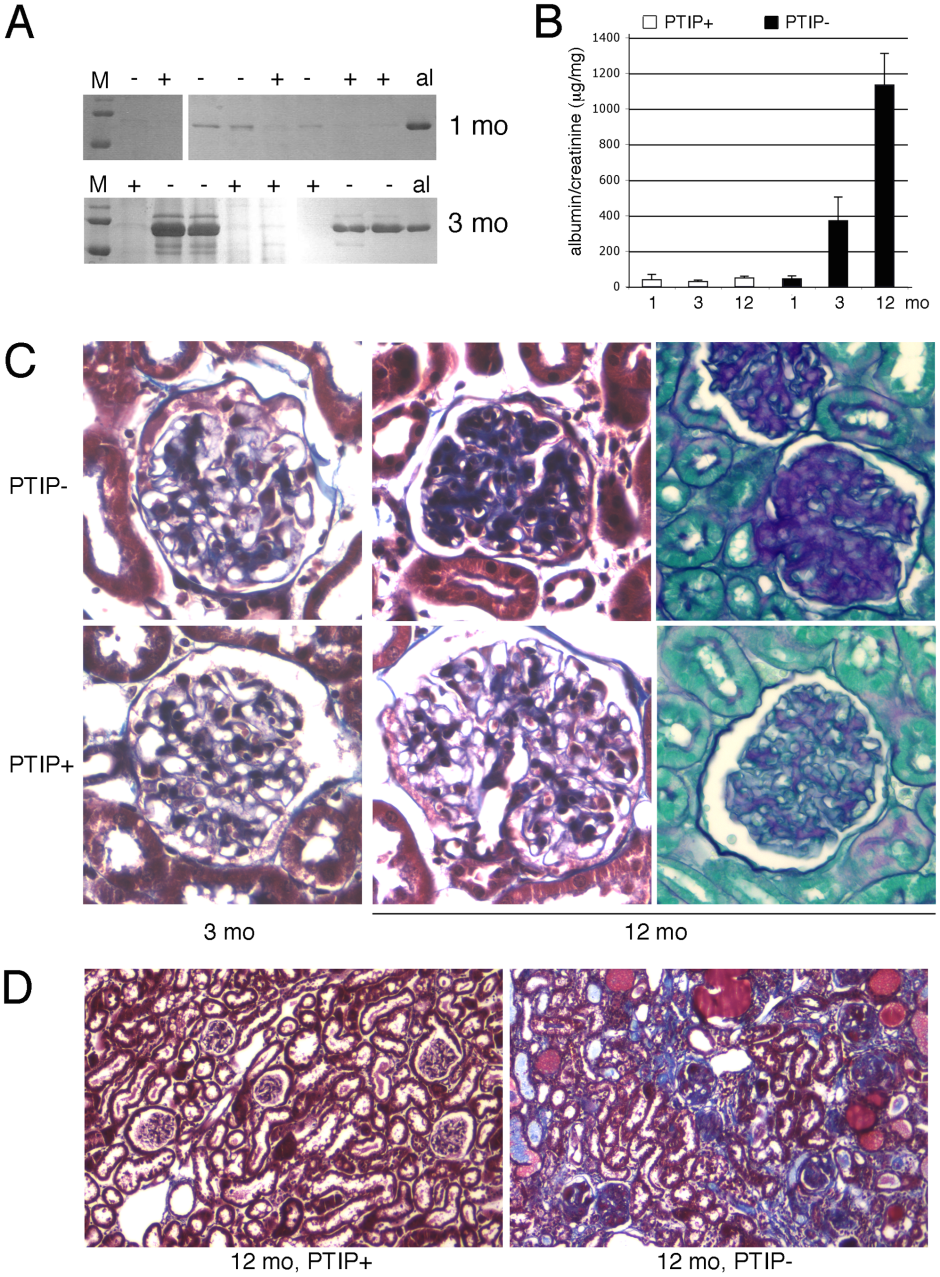
Chronic Glomerular Disease in PTIP− Kidneys. A) Coomassie blue staining of SDS/PAGE gels of urine samples from PTIP+ and PTIP− mice at 1 month and 3 months. Mouse albumin (al) is shown as a control. B) Urine albumin to creatinine ratios (ACR) as measured at 1, 3, and 12 months of age in PTIP+ and PTIP− animals. C) Histological sections from kidneys at 3 and 12 months. Representative glomerular sections were stained with Masson's Trichrome (3 and 12 months) or Periodic Acid-Shiff (12 months). Significant matrix deposition was observed in 12 months old PTIP− glomeruli. D) Low power view of a kidney section at 12 months of age shows tubulointerstitial fibrosis, protein filled cysts, and glomerular sclerosis in PTIP− animals.

Podocytes play a critical role in the establishment and maintenance of the glomerular filtration barrier. Interdigitated podocyte foot processes cover the glomerular basement membrane and form specialized junctions, called slit diaphragms, which create a highly selective barrier that filters small and negatively charged proteins and solutes from the blood to the urinary space. Damage to or loss of podocytes impairs the filtration barrier and results in increased rates of excretion of high molecular weight proteins, such as albumin, into the urine. Thus, we checked mice for proteinuria beginning at 1 month of age (Figure 2A). At 1 month, low levels of albumin were detected in the urine but these were not significantly different between PTIP+ and PTIP− animals. However, by 3 months of age the PTIP− mice showed significantly higher levels of albumin in the urine and these levels increased further at 6 and 12 months. The urine albumin to creatinine ratio (ACR) provides a quantitative assay that correlates with filtration barrier integrity. No significant differences were observed at 1 month (Figure 2B). However, by 3 and 12 months, ACR were 10 and 30 fold higher respectively in urines of PTIP− animals compared to PTIP+ mice. Mice that carried the *Cre* ^NPHS2^ transgene in a *Paxip1^+/+^* or a *Paxip1^fl/+^* genetic background did not show any renal abnormalities at 12 months (data not shown), consistent with many published reports that have used this particular Cre driver strain [25]–[28].

Renal pathology was characterized by light microscopy at 1, 3, and 12 months of age. Standard Masson's Trichrome and Periodic-Acid-Shiff stainings revealed significant sclerosis and matrix deposition in 12 month old glomeruli from PTIP− animals (Figure 2C). However, 3 month old kidneys did not show significant differences for most glomerular sections, at the light microscopy level, although evidence of limited matrix expansion could be observed in a small number of glomeruli of PTIP− kidneys. In 12 month old kidneys, significant interstitial fibrosis and protein filled cysts were also observed (Figure 2D). These are likely to be secondary effects due to the glomerular pathology.

**Figure 3:**
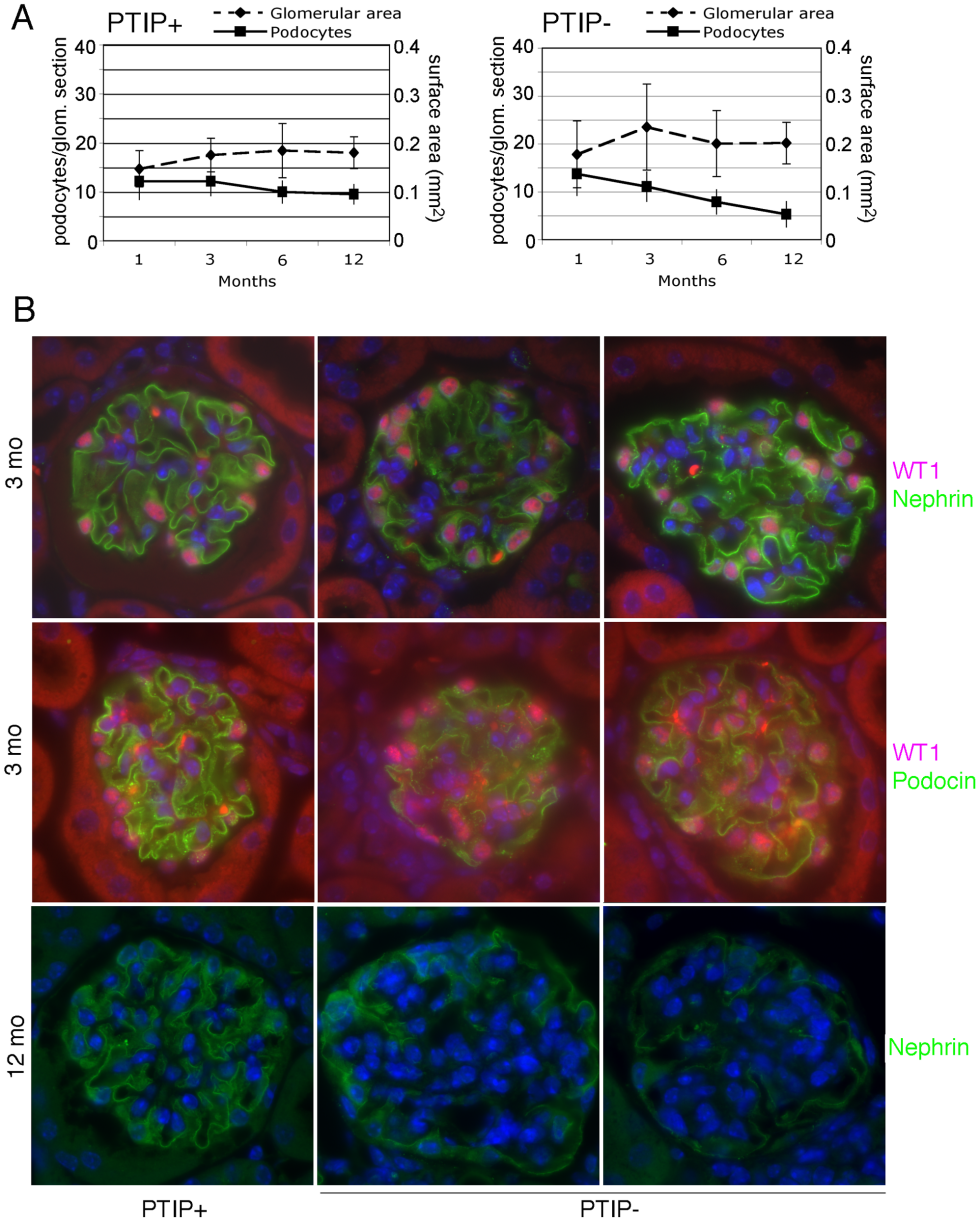
Podocyte Viability and Glomerular Morphology. A) After immunostaining with WT1 and Nephrin antibodies, podocyte nuclei were counted in mid-cross sections through glomeruli whose vascular and proximal tubular poles were visible. Glomerular surface area for mid-cross sections was measured by morphometry and is expressed in relative units. B) Immunostaining for WT1 (pink) and Nephrin (green) at 3 months of age shows little significant difference between PTIP+ and PTIP− glomeruli. However, Podocin staining (green, lower panels) appears less and discontinuous in PTIP− glomeruli. Nuclei were counterstained with DAPI. By 12 months, large regions cleared of Nephrin positive staining were evident within the glomerular tufts of PTIP− animals.

Glomerular pathology and increased albuminuria can be the direct result of podocyte death [29]. Thus, we used a variety of markers to characterize the glomerular architecture and the numbers of podocyte cells at various ages to insure that the phenotype of the PTIP− mice was not just the result of early podocyte cell death. Immunostaining with WT1, Nephrin, and Podocin antibodies enabled us to determine the podocyte numbers, as average per mid-cross section, and to indirectly assess the integrity of the slit diaphragm (Figure 3). The number of WT1 positive podocytes was not significantly different between PTIP+ and PTIP− glomeruli at 1 or 3 months of age. At 6 months, PTIP− glomeruli had slightly fewer podocytes and by 12 months, the number of podocytes was half that of the PTIP+ littermates. Immunostainings for podocyte markers such as WT1, Nephrin, and Podocin did not reveal dramatic differences at 1 or 3 months, despite the increase in proteinuria, although some discontinuous staining could be seen with Podocin antibodies in PTIP− glomeruli (Figure 3B). Consistent with this data, TUNEL staining for apoptosis did not reveal differences between PTIP+ and PTIP− kidneys at 1 or 3 months of age (data not shown). Thus, the breakdown of the filtration barrier was not due to simple podocyte depletion at these early times. However by 12 months of age, the extensive network of Nephrin staining was partially depleted in PTIP− glomeruli (Figure 3B).

**Figure 4:**
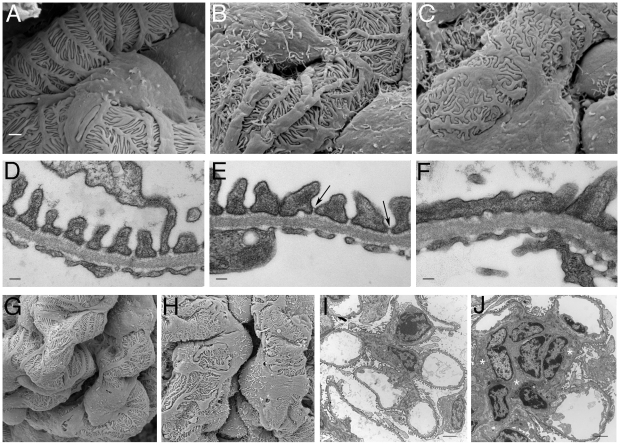
Ultrastructural Analysis of PTIP− Kidneys. Podocytes of PTIP− mice showed progressive foot process disorganization and effacement, as observed by scanning (A–C, G, H) and transmission (D–F, I, J) electron microscopy. Podocyte foot processes of 3-month-old PTIP+ mice were regularly interdigitated (A, D, G), whereas those of age-matched PTIP− podocytes (B, C, E, F, H) displayed varying degrees of disorganization (B, E) and effacement (C, F). Note that slit diaphragms could still be observed between foot processes during the early stages of disorganization (E, *arrows*). G–J) In addition to the foot process alterations, capillary loop deformation/enlargement (H, J) and mesangium expansion (J, *asterisks*) were observed in glomeruli of 12-month-old (G, H) and 3-month-old (I, J) mice analyzed by EM. Scale bars: (A–C) 1 µm; (D–F) 100 nm; (G–J) 2 µm.

At the light microscopy level, the effects of PTIP loss on glomerular architecture seemed minimal at 3 months of age, yet the levels of albumin in the urine suggested significant functional defects. Thus, we utilized scanning and transmission electron microscopy to characterize the podocytes at the ultra structural level (Figure 4). Scanning electron micrographs revealed disorganized foot processes at 3 months. While PTIP+ podocytes had regularly arrayed tertiary foot-processes that were almost parallel (Figure 4A), the PTIP− podocyte foot processes were much more irregular and flattened. The parallel pattern of interdigitation was clearly different and resembled a jigsaw puzzle with random patterning (Figure 4B, 4C). Transmission electron micrographs at 3 months also revealed that the slit-diaphragms were not evenly spaced and fusion of foot processes was frequent (Figure 4D–4F). By 12 months, the remaining podocytes in the PTIP− kidneys were broader, flatter and displayed significant fusion or effacement (Figure 4G, 4H), consistent with the high levels of albumin detected in the urine. These data demonstrate that the initial glomerular phenotype in PTIP− kidneys is due primarily to differences in podocyte foot process morphology, which occurs prior to the loss of cell bodies.

### Alteration of the Gene Expression Program Precedes the Disease Phenotype

**Figure 5:**
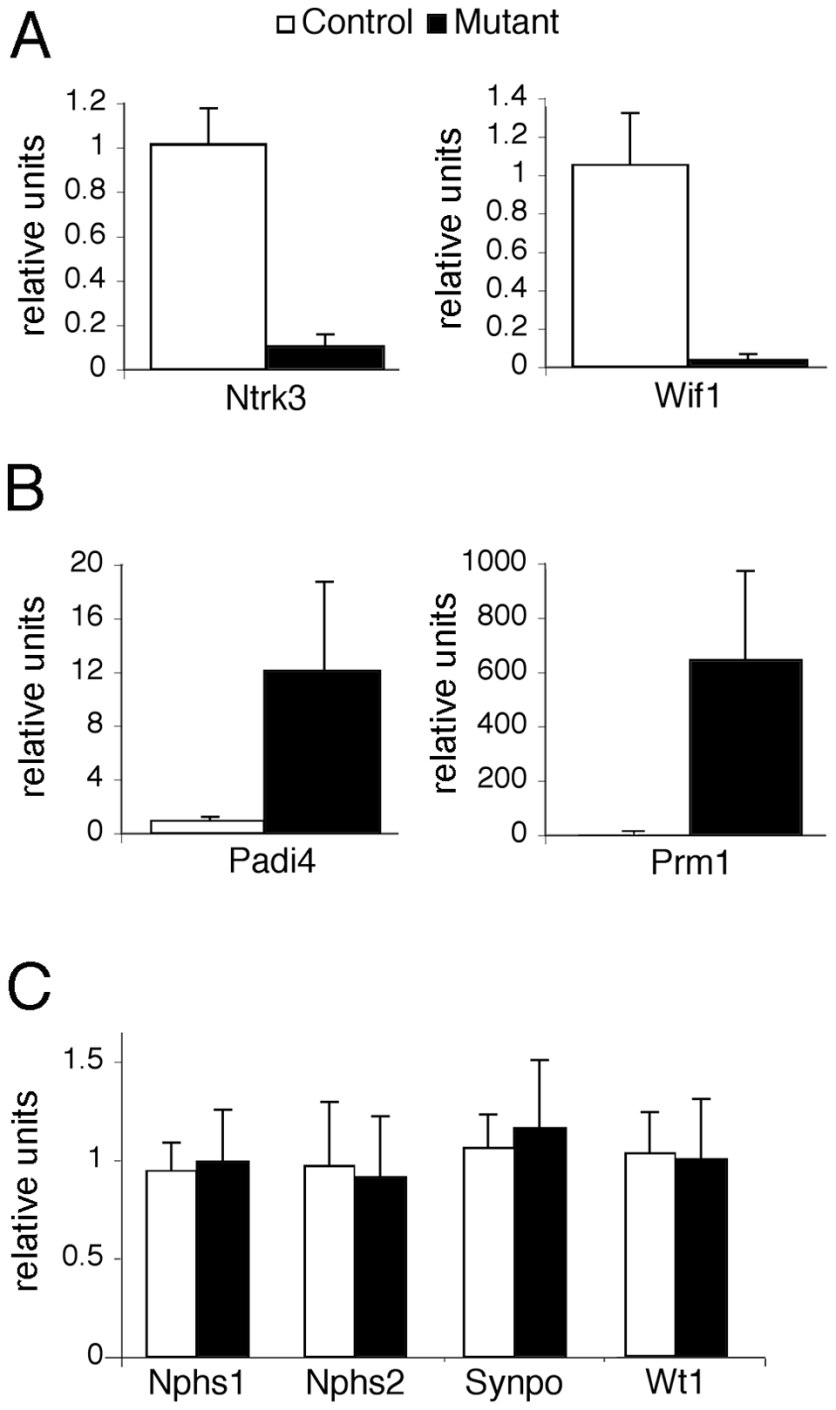
Gene Expression in the Glomerulus. Real-time qRT-PCR for the indicated genes was performed on total RNA isolated from glomerular preparations. A) Confirmation of two genes that are down-regulated in PTIP− (black) kidneys compared to controls PTIP+ (open) kidneys. B) Confirmation of two genes that are up-regulated in PTIP− kidneys compared to controls. C) Expression levels of podocyte marker genes in PTIP+ and PTIP− glomerular preparations.

Alterations in cellular phenotypes could be the result of changes in the transcriptional program of PTIP− podocytes. Thus, we prepared RNA from glomeruli enriched fractions at 1 month of age, prior to the onset of any significant phenotype, and assayed for gene expression changes by Affymetrix microarrays. We compared glomerular RNA preps from 10 independent PTIP− animals and 8 PTIP+ littermates at 1 month of age. The data were highly consistent and indicated both gain and loss of gene expression in the PTIP− kidneys (Table 1 and Table 2). The entire dataset can be accessed at the Gene expression Omnibus (GSE17709). Expression changes were confirmed by quantitative RT-PCR for selected genes (Figure 5). Among the genes increased was *Protamine1* (*Prm1*), which is not normally expressed in podocytes or other somatic cells but is found only in spermatids where it is essential for chromatin condensation and fertility [30], [31]. The changes in RNA expression observed were surprising and did not correspond to any common pathways. In fact, the podocyte-specific genes that are known to function in cell viability and slit diaphragm integrity were largely unchanged (Table S1 and Figure 5C). The data suggest that loss of PTIP in podocytes alters the transcriptional program to affect a limited number of genes whose functions in the podocytes have not been previously characterized.

**Table 1:** Genes Up-Regulated in PTIP− Podocyte.

Probe	Symbol	Description	UniGene	p-value	Fold Change *
1439379	Prm1	protamine 1	Mm.42733	0	6.38
1418398	Tspan32	tetraspanin 32	Mm.28172	0	2.92
1422760	Padi4	peptidyl arginine deiminase, type IV	Mm.250358	0.001	1.9
1433744	Lrtm2	leucine-rich repeats and transmembrane domains 2	Mm.121498	0	1.89
1433529	E430002G05Rik	RIKEN cDNA E430002G05 gene	Mm.28649	0	1.77
1436329	Egr3	early growth response 3	Mm.103737,	0	1.74
1449071	Myl7	myosin, light polypeptide 7, regulatory	Mm.46514	0.001	1.68
1419527	Comp	cartilage oligomeric matrix protein	Mm.45071	0	1.58
1419487	Mybph	myosin binding protein H	Mm.379067	0.001	1.3
1431991	2410004P03Rik	RIKEN cDNA 2410004P03 gene	Mm.159048	0	1.26
1430062	Hhipl1	hedgehog interacting protein-like 1	Mm.36423	0.004	1.19
1453228	Stx11	syntaxin 11	Mm.248648	0.003	1.16
1416077	Adm	adrenomedullin	Mm.1408	0	1.14
1457780	Stx11	syntaxin 11	Mm.248648	0.001	1.1
1434984	6330514A18Rik	RIKEN cDNA 6330514A18 gene	Mm.17613	0.004	1.08
1453152	Mamdc2	MAM domain containing 2	Mm.50841	0.012	1.05
1435830	5430435G22Rik	RIKEN cDNA 5430435G22 gene	Mm.44508	0.002	1.01
1439761	D830026I12Rik	RIKEN cDNA D830026I12 gene	Mm.136046	0.008	1

*log2 scale.

**Table 2:** Genes Down-Regulated in PTIP− Podocytes.

Probe	Symbol	Description	UniGene	p-value	Fold Change *
1425425	Wif1	Wnt inhibitory factor 1	Mm.32831	0	−4.37
1441491	A330068G13Rik	RIKEN cDNA A330068G13 gene	Mm.227543	0	−3.68
1433825	Ntrk3	neurotrophic tyrosine kinase, receptor, type 3	Mm.33496	0	−3.09
1446622	A330068G13Rik	RIKEN cDNA A330068G13 gene	Mm.227543	0	−2.14
1452779	3110006E14Rik	RIKEN cDNA 3110006E14 gene	Mm.23960	0	−1.57
1452416	Il6ra	interleukin 6 receptor, alpha	Mm.2856	0	−1.55
1420903	St6galnac3		Mm.440929	0	−1.53
1450309	Astn2	astrotactin 2	Mm.445312	0	−1.53
1433939	Aff3	AF4/FMR2 family, member 3	Mm.336679	0	−1.53
1437403	Samd5	sterile alpha motif domain containing 5	Mm.101115	0.001	−1.48
1429896	5830408B19Rik	RIKEN cDNA 5830408B19 gene	Mm.291322	0	−1.35
1455296	Adcy5	adenylate cyclase 5	Mm.41137	0	−1.3
1431946	Necab3	N-terminal EF-hand calcium binding protein 3	Mm.143748	0	−1.29
1434777	Mycl1	v-myc myelocytomatosis viral oncogene homolog 1	Mm.1055	0	−1.26
1419139	Gdf5	growth differentiation factor 5	Mm.4744	0.001	−1.25
1441559	LOC627626	similar to CG11212-PA	Mm.390999	0.003	−1.25
1441667	Smyd1	SET and MYND domain containing 1	Mm.234274	0	−1.23
1423561	Nell2	NEL-like 2 (chicken)	Mm.3959	0.016	−1.18
1450501	Itga2	integrin alpha 2	Mm.5007	0	−1.17
1435832	Lrrc4	leucine rich repeat containing 4	Mm.443660	0	−1.11
1455188	Ephb1	Eph receptor B1	Mm.22897	0.046	−1.11
1455888	Lingo2	leucine rich repeat and Ig domain containing 2	Mm.132507	0.007	−1.05
1426960	Fa2h	fatty acid 2-hydroxylase	Mm.41083	0	−1.04
1453841	2310050P20Rik	RIKEN cDNA 2310050P20 gene		0.033	−1.01
1421207	Lif	leukemia inhibitory factor	Mm.4964	0	−1

*log2 scale.

### PTIP Deletion Affects *Ntrk3* Expression and Histone Methylation

**Figure 6:**
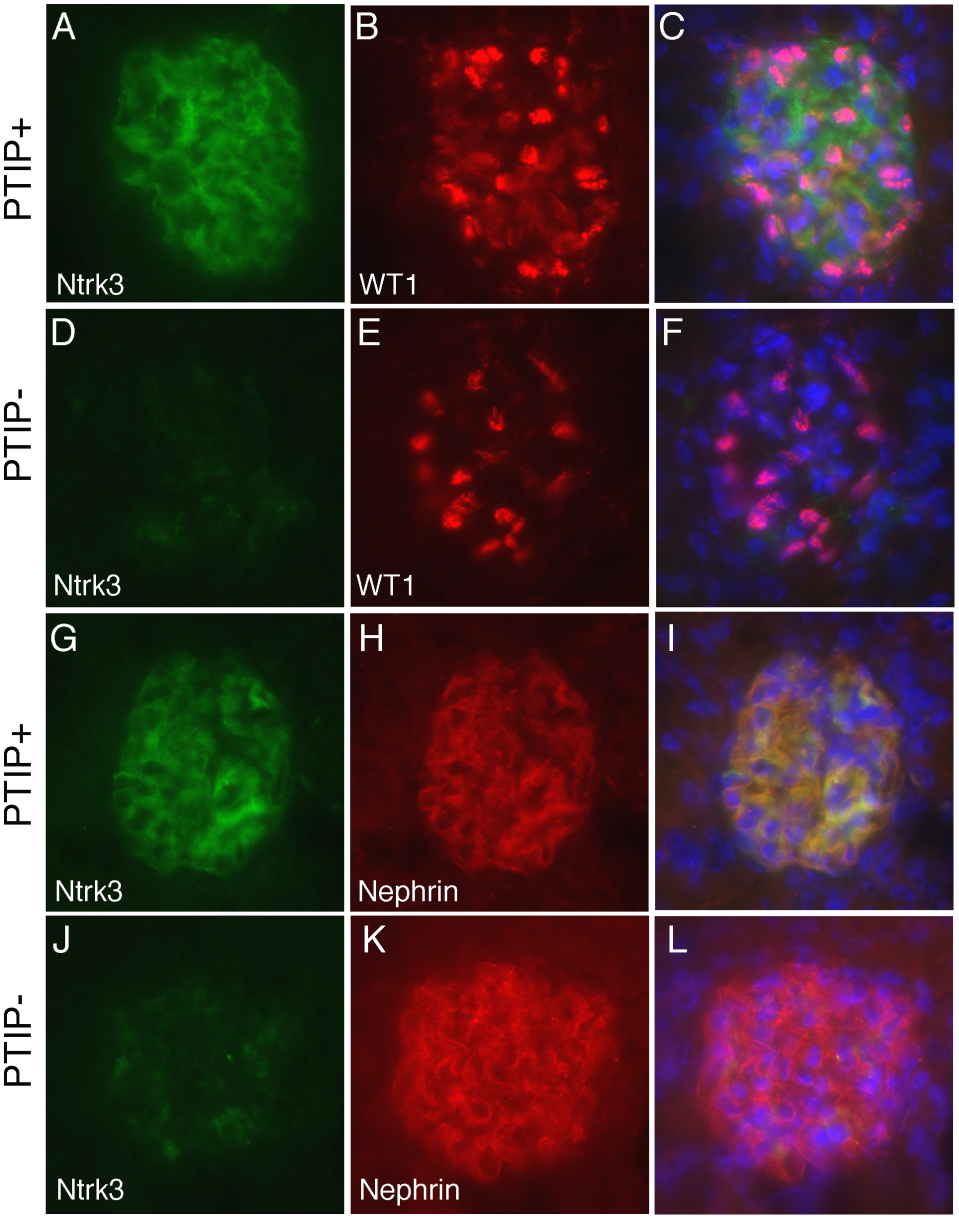
Ntrk3 in the Glomerulus. Fresh frozen tissues were sectioned and fixed in methanol followed by immunostaining with goat anti-Ntrk3, rabbit anti-WT1, or rabbit anti-Nephrin, as indicated. PTIP+ sections (A–C, G–I) showed strong Ntrk3 staining in all glomeruli, in a pattern similar to Nephrin. The PTIP− kidney sections (D–F, J–L) showed much lower levels of Ntrk3 protein in glomeruli. All micrographs were taken at manually set, equal exposures. Right panels (C, F, I, L) are overlays of Ntrk3 and WT1 or Ntrk3 and Nephrin and are counterstained with DAPI (blue) to visualize all cell nuclei.

Among the most interesting genes whose expression was down regulated in PTIP− kidneys was the neurotrophic tyrosine kinase receptor type 3 (*Ntrk3*, formerly called *TrkC*), whose expression in podocytes had not been previously described. The *Ntrk3* gene encodes two proteins that recognize neurotrophin 3 (NT-3) and functions in axon guidance and innervation and in cardiac development [32]–[34]. Ntrk3 promotes axon outgrowth and guidance, presumably through actin based extension and retraction of cellular processes [35]. Given that podocyte foot processes are also actin based and may require some type of guidance, we examined the role of Ntrk3 further. Quantitative RT-PCR confirmed that *Ntrk3* expression was down approximately 10 fold in glomerular preps from PTIP− compared to PTIP+ animals (Figure 5A). We also examined Ntrk3 levels in kidneys by co-immunostaining kidney sections with Ntrk3, WT1 and Nephrin antibodies (Figure 6). At 3 months of age, Ntrk3 could be seen in glomeruli of PTIP+ kidneys, however the staining intensity in PTIP− kidneys was severely reduced in almost every glomerulus examined (Figure 6D, 6J). Some slight filamentous staining remained in the PTIP− glomeruli, but the overall intensity was markedly different. In PTIP+ glomeruli, Ntrk3 staining was remarkably similar to Nephrin (Figure 6G–6I). However, Nephrin staining intensity was unaffected in PTIP− glomeruli even though Ntrk3 was much lower (Figure 6J–6L). The *Ntrk3* expression in glomerular preps and its decrease in the PTIP− kidneys suggested a function in foot process growth, guidance, and/or pattern formation.

**Figure 7:**
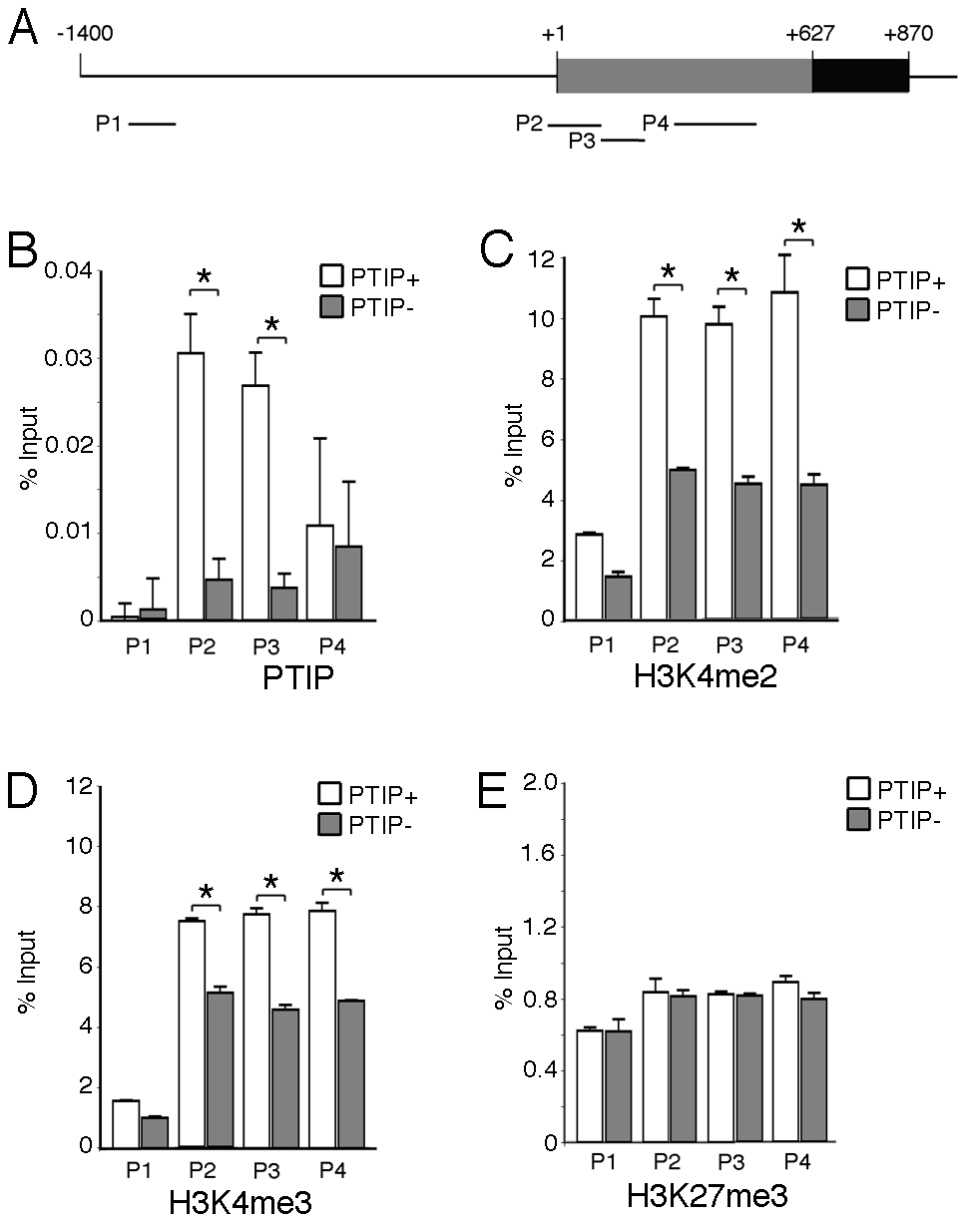
Chromatin Immunoprecipitation (ChIP) at the *Ntrk3* Locus. A) Schematic of the sequences surrounding the first *Ntrk3* exon , the transcription start site (+1) and the ATG start codon (+627) are indicated. The positions of the four primers used for PCR analyses of the immunoprecipitated chromatin are shown. B) ChIP experiment using anti-PTIP antibodies and chromatin from whole glomeruli enriched from PTIP+ (open bars) and PTIP− (grey bars) kidneys. C) ChIP experiment as in B but with anti-H3K4me2 antibodies. D) ChIP experiment as in B but using anti-H3K4me3 antibodies. E) ChIP experiment as in B but using anti-H3K27me3 antibodies. For B–E, all values are expressed as the mean of 3 replicates; error bars are one standard deviation. Statistically significant differences are indicated (*P<0.05).

In order to more directly link PTIP to the *Ntrk3* locus, we designed chromatin immunoprecipitation experiments to examine the presence of PTIP and the changes in histone methylation patterns around the transcription initiation site (+1) of *Ntrk3* (Figure 7). Chromatin was prepared from whole glomerular preps from PTIP+ and PTIP− kidneys, which also included mesangial and endothelial cells. Despite the presence of other cell types in the glomerular chromatin, we were able to detect a 5–6 fold decrease in PTIP localization to sequences around the start site of *Ntrk3* transcription when comparing PTIP+ to PTIP− chromatin (Figure 7B). No significant amount of PTIP was detected further upstream (−1200), nor did we see a significant difference, between PTIP+ and PTIP− chromatin, in PTIP localization within the 5′ UTR of exon 1 (Figure 7B, P4 site). Clear differences in H3K4me2 were also measured, with an approximately 50–60% decrease in PTIP− chromatin with primer pairs P2–P4, but not with P1 at −1200 (Figure 7C). Similarly, H3K4me3 levels were also decreased in PTIP− chromatin at P2–P4 but not at P1 (Figure 7D). We also examined changes in Polycomb mediated epigenetic silencing marks using an antibody against H3K27me3 (Figure 7E), which appeared unchanged at all sites examined. These data demonstrate recruitment of PTIP to the promoter region of *Ntrk3* in normal glomeruli.

### *Ntrk3* Mutants Have Podocyte Foot Process Defects

**Figure 8:**
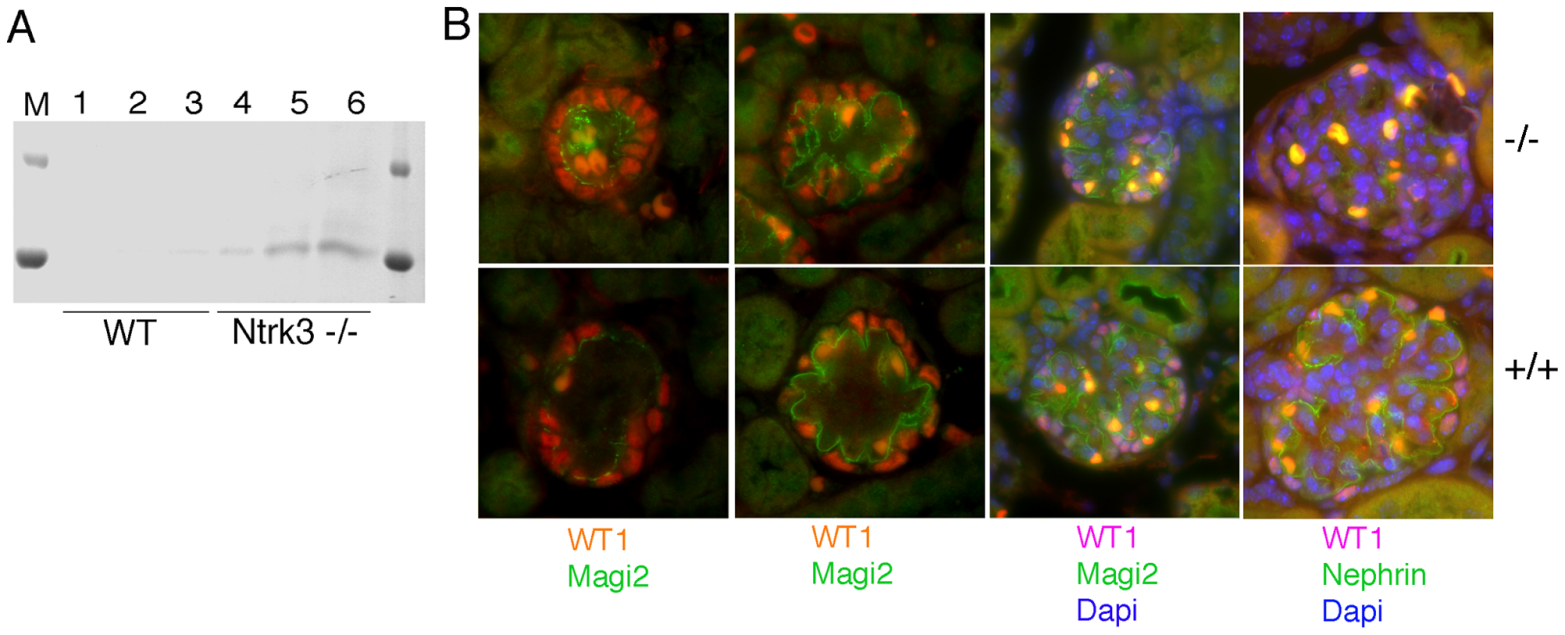
Analysis of Ntrk3 Mutant Kidneys. A) Comassie stained SDS/PAGE gels of urine collected from 4 day old *Ntrk3^−/−^* and wild-type littermates. B) Immunostaining of 4 day old kidneys from wild-type and *Ntrk3^−/−^* kidneys as indicated. From left to right, glomeruli are shown at increasingly older stages of development. Note discontinuous Magi2 staining and reduced Nephrin staining in older glomeruli of *Ntrk3^−/−^* kidneys compared to control littermates.

In order to determine if the loss of Ntrk3 alone would impact normal glomerular patterning, we examined homozygous *Ntrk3* mutant mice. The *Ntrk3* mutants die shortly after birth due to cardiac and neuromuscular defects; however their kidneys had not been studied previously. Therefore, we collected urine and kidney tissue for light and electron microscopy from 3–4 day old *Ntrk3* mutants and littermates. At three days post partum, *Ntrk3* mutants were small and sickly. Higher levels of albumin could be observed in the urines of *Ntrk3^−/−^* pups (Figure 8A), compared to control littermates, although this could be due to delayed or arrested kidney development. Glomerular development was examined in kidney sections of 4 day old newborns (Figure 8B). At this time, nephrons are still undergoing development and glomeruli at the periphery are just beginning to form whereas cortical glomeruli closer to the medulla are already fully functional. The tight junction protein Magi2 specifically localizes to podocyte cell junctions and exhibited altered patterning in *Ntrk3* mutant kidneys, with discontinuous staining and excessive looping of the developing tuft. In mature glomeruli, Nephrin staining was reduced and patchy in the *Ntrk3* mutants. The number of podocytes did not seem affected in the *Ntrk3^−/−^* mice at this time.

**Figure 9:**
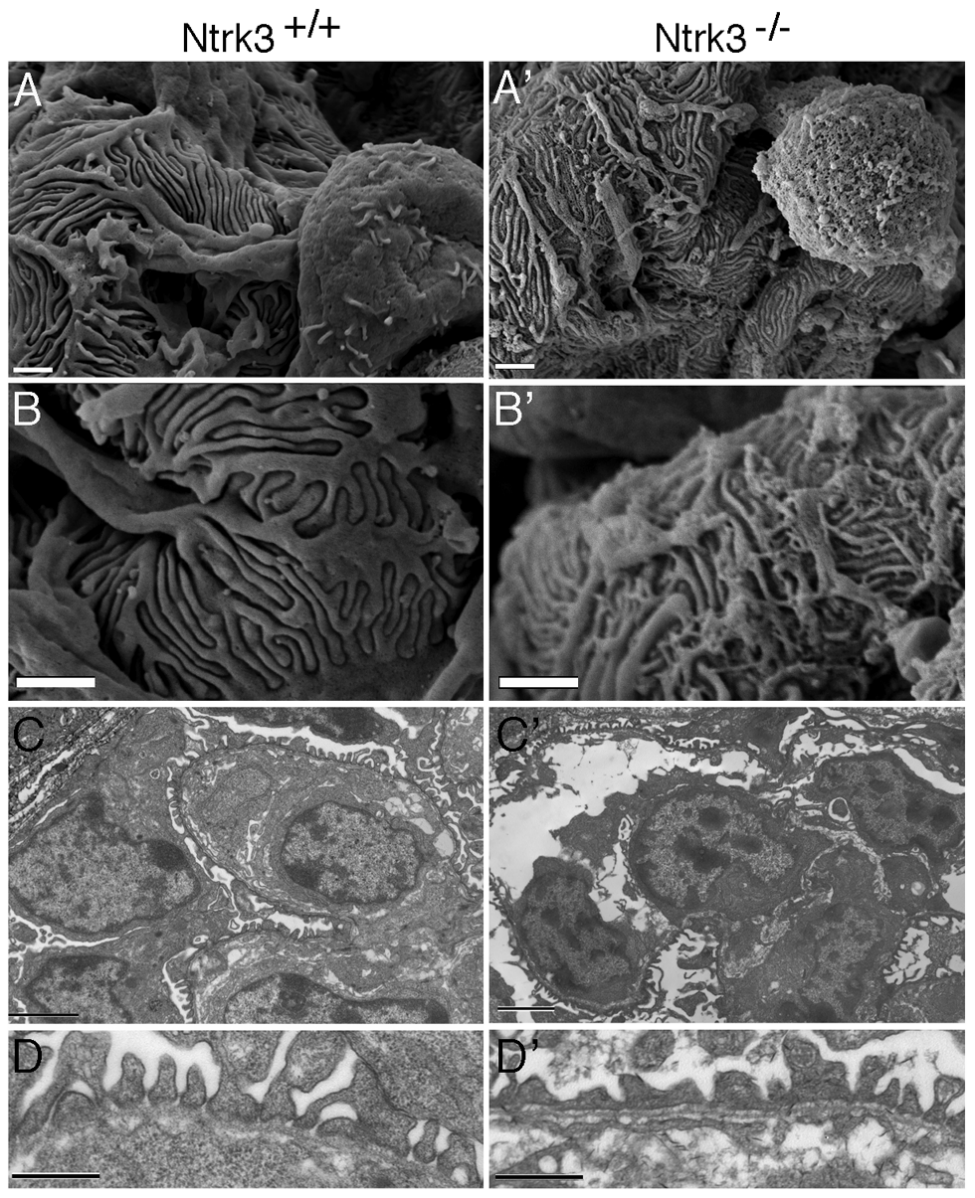
Ultrastructural Analysis of *Ntrk3* Mutant Kidneys. Kidneys from *Ntrk3^−/−^* (′) and control littermates at 4 days of age were examined by scanning (A, B) and transmission electron microscopy (C, D). A, B) Note the disorganized patterning and irregularly shaped primary and secondary foot processes. C, D) Note the fusion of foot processes and the lack of well-spaced slit diaphragms in *Ntrk3* mutants in D. Scale bars are 1 µm in A and B, 2 µm in C, and 500 nm in D.

Ultra structural analysis of *Ntrk3* mutant kidneys revealed podocyte patterning defects both by scanning and transmission EM (Figure 9). At 4 days post-partum, we examined the most mature glomeruli, those located closest to the medullary zone. Podocyte foot processes from *Ntrk3^−/−^* mice exhibited disorganized secondary and tertiary processes that crisscrossed randomly over capillary vessels and were poorly interdigitated (Figure 9A′, 9B′). Few sections showed the characteristic spacing indicative of the slit diaphragms at the glomerular basement membranes (Figure 9D′). These data suggest a critical role for *Ntrk3* in the fine patterning events of secondary and tertiary foot process formation and interdigitation.

## Discussion

In this report, we utilized a conditional deletion to ask whether the PTIP dependent H3K4 methylation function is required in a terminally differentiated cell type, to maintain its differentiated state and its cell-type specific transcriptional program. Using the glomerular podocyte cell as a model, we show that deletion of PTIP results in subtle changes in gene expression patterns that ultimately lead to a slowly progressing disease state. These data support a model in which the gross stability of the differentiated state or podocyte cell survival, at least in the short term, does not depend on the PTIP/KMT complex, as many of the podocyte specific genes examined were unchanged in the absence of PTIP. Rather, the loss of PTIP was more subtle and revealed unexpected changes in a small number of genes and ultimately led to a chronic disease phenotype resembling glomerular sclerosis. Typical characteristics of chronic glomerular disease were present, including microalbuminuria, podocyte foot process fusion or effacement, remodeling of the filtration barrier, and increased extracellular matrix deposition.

Methylation of histone H3 at lysine 4 correlates with gene expression and is thought to regulate cellular identity by establishing and maintaining a stable epigenetic state. The PTIP protein is part of an H3K4 methyltransferase complex that includes the mammalian Trithorax homologues KMT2B and/or KMT2C [10], [11], [13], [16]. Previous studies in flies and mice demonstrated reduced H3K4 methylation in *Paxip1* mutants and severe early lethal phenotypes. In the mouse, complete loss of PTIP protein results in developmental arrest just after gastrulation [14], a phenotype more severe than any individual mouse KMT2 family gene mutation [12], [36], [37], whereas a hypomorphic *Paxip1* allele is lethal later in development [38]. In flies, maternal and zygotic *ptip* null embryos are embryonic lethal and fail to express many segmentation genes [17]. In mouse embryonic stem cells, PTIP protein is required for normal levels of H3K4 methylation and for maintaining pluripotency in cell culture [15], whereas in embryonic fibroblasts PTIP is required for adipocyte differentiation [16]. All of these findings suggest that a PTIP H3K4 methyltransferase complex is needed for differentiation of stem cells and progenitor cells in development. However in terminally differentiated cells, the requirement for active H3K4 methylation may be different and the lack of cell division may abrogate the need for de novo methylation. Our results suggest that PTIP must still function in some non-dividing cells, perhaps as part of a maintenance complex, as overall levels of H3K4 methylation were reduced and activation and suppression of a small number of genes was affected.

The mature podocyte is generally believed to be a non-dividing cell type, as classic cell BrdU labeling experiments do not mark this population over time [39]. However, more recent genetic lineage tracing experiments suggest that there is a population of parietal epithelial cells at the vascular pole of the Bowman's capsule that can replenish podocytes over time [40], [41]. This replacement of podocytes appears slow under normal conditions, but may be especially critical in cases of glomerular injury. In our animal model, we would expect any podocyte replacement to also delete the *Paxip1* gene once expression of the Cre driver is activated. Given that we do not see significant loss of podocytes until at least 6 months of age, it may be that alterations in the transcriptional profile are not lethal. Rather, loss of podocytes may be the result of the damaged filtration barrier, the increase in the mesangium, and the general environment of the glomerulus in older mice. Alternatively, if podocyte replacement is accelerated in our model, it may be that by 6 months the ability of parietal cells to replenish the podocyte population is exhausted. In either case, the effects of manipulating the H3K4 methylation pathway is more apparent in older mice, suggesting a critical role for such epigenetic pathways in aging cells and tissues.

The changes in gene expression observed in response to PTIP deletion are surprising in that most of the well-characterized podocyte-specific genes appear unaffected. However, changes include both activation and suppression of previously uncharacterized genes in the podocytes. Activation of the *Prm1* gene in PTIP− kidneys is unusual as this gene has only been associated with sperm maturation and is thought to encode a unique chromatin binding protein [31], [42]. Activation of the *Padi4* gene could impact gene expression by deimination of arginines in the histone H3 tail, which prevents methylation [43]. The impact of increased Padi4 is likely to be complex as arginine methylation can correlate with gene activation or repression, depending on the context and specific residues.

The most compelling gene affected in PTIP− podocytes was *Ntrk3*, whose expression in the glomerulus had not been previously characterized. The reduction of *Ntrk3* expression in PTIP− kidneys and the phenotype of *Ntrk3^−/−^* newborn kidneys suggest that this receptor is critical for tertiary foot process pattern formation. The podocyte is a highly specialized cell with a complex network of processes that cover the glomerular basement membrane. The large primary processes are microtubule containing structures, whereas the tertiary, interdigitated foot processes contain actin microfilaments [44]. Adjacent foot processes are connected through a specialized junctional complex, called the slit diaphragm, which is essential for maintaining a functional filtration pore. Some of the essential proteins in the slit-diaphragm, such as Nephrin, Podocin, and Neph1 are well characterized and mutations are associated with severe nephrotic syndromes [45]. Yet, how foot process outgrowth is regulated and maintained is not clear. Our data suggests that Ntrk3, and by inference its ligand NT–3, may be important for foot process growth and patterning. NT-3 is known to promote neuronal axon guidance by stimulating actin polymerization and lamellipodia formation [46], [47]. In cultured neuronal cells, NT-3 promotes localization of β-actin mRNA to the growth cones to stimulate motility and chemotaxis [48], [49]. Podocytes express many proteins known to function in neurite outgrowth, such as semaphorins, neuropilins, and ephrins. A recent report even describes the release and up-take of glutamate containing synaptic-like vesicles by podocytes [50]. Furthermore, foot processes are dynamic and can retract quickly in response to polyamines like protamine sulfate [51], [52]. This raises the possibility that sensing mechanisms are required for rapid actin dynamics; such mechanisms may be common to both podocytes and neurons. Still, reduction of Ntrk3 alone is unlikely to cause the phenotypic changes in PTIP− podocytes over time, as other genes whose functions are not well understood are also impacted.

Histone methylation by Trithorax or Polycomb complexes can imprint positive and negative epigenetic marks on chromatin during development. More recently, histone methyltransferases have been associated with cancer and other disease states. However, in many cases it is not clear whether changes in the expression of epigenetic modifiers are the cause or the result of disease progression. The results presented here suggest that mutations in an epigenetic pathway, which result in alterations of H3K4 methylation patterns, can lead to a chronic disease through subtle changes in gene expression patterns. This implies a direct function for HMTs in maintaining gene expression and the differentiated state in healthy organisms.

## Methods

### Animals

Mice carrying the *Paxip1* null (*Paxip1* ^−^) and floxed (*Paxip1* ^fl^) alleles were previously described and genotyped as indicated [14], [53]. To obtain the specific deletion of the *Paxip1* ^fl^ allele in glomerular podocytes, these mice were crossed with the previously characterized 2.5P-Cre mice [24], [25], which express the Cre recombinase under the control of the human *NPHS2* promoter (*Cre* ^NPHS2^). Among the next generations, mice carrying the Cre allele (*Paxip1* ^fl/fl^:*Cre* ^NPHS2^ and *Paxip1* ^fl/−^:*Cre* ^NPHS2^ mice) were considered as conditional null mutants (PTIP−), whereas littermates that did not express the Cre recombinase were used as controls (PTIP+). All animal procedures were approved by the University Committee on Use and Care of Animals (UCUCA) of the University of Michigan and performed in compliance with ULAM recommendations.

### Antibodies

Rabbit polyclonal antibodies used to detect Nephrin (1∶1000) and Podocin (1∶500) were kindly provided by L.B. Holzman (University of Pennsylvania, Philadelphia, PA). Chicken anti-PTIP was described previously [54]. Additional antibodies were commercially available: mouse clone 6F-H2 anti-WT1 (1∶1000, DAKO, Carpinteria, CA), anti-H3K4me3 and anti-H3K27me3 (AbCam, Cambridge, MA), anti-Magi2 (Sigma-Aldrich, St. Louis, MO), anti-Ntrk3 (AF1404, R & D Systems, Minneapolis, MN), Alexa Fluor 488 F(ab′)2 fragment of goat anti-rabbit IgG, Alexa Fluor 568 F(ab′)2 fragment of goat anti-mouse IgG, Alexa Fluor 488 donkey anti-goat IgG (1∶500; Molecular Probes, Life Technologies, Carlsbad, CA).

### Urine Collection and Analysis

Mice had access to a standard breeder chow (Purina 5008) and water *ad libitum*. Urine was collected early in the afternoon for three consecutive days from individual mice at 1, 3, 6 and 12 months of age and stored frozen until use. After thawing, 2 µL urine was run on a SDS-PAGE and stained with Coomassie Blue to test for the presence of proteins/albumin, using recombinant mouse albumin (Sigma-Aldrich, St. Louis, MO) as a control. Quantitative assessment of urine albumin and creatinine concentrations were determined by ELISA using the Albuwell M and Creatinine Companion kits (Exocell Inc., Philadelphia, PA).

### Specimen Preparation for Microcopy Analyses

Mice at 1, 3, 6, and 12 months of age were sacrificed and their kidneys were perfused, fixed, and processed for histology, indirect fluorescence and electron microcopy analyses. Briefly, mice were anesthetized by intraperitoneal injection of 40 mg/kg sodium pentobarbital and prepared for systemic perfusion. A saline solution was first injected through the abdominal aorta to the entire mouse body at a pressure of approximately 70 mmHg as previously described [55]. As soon as the general bloodstream had been cleared, a solution of 4% paraformaldehyde in PBS was substituted. It was left to perfuse at the same flow conditions for approximately 10 minutes. Kidneys were removed, decapsulated, cut into pieces, and incubated for 2 additional hours in the appropriate fixative solution before being processed for histology, indirect immunofluorescence, and electron microscopy.

### Histology and Indirect Immunofluorescence

Kidneys were fixed in 4% paraformaldehyde, embedded in paraffin, sectioned at 5 microns, and stained with Periodic Acid Shiff or Masson Trichrome. For immunofluorescence analyses with Nephrin, PTIP, WT1 and Magi2, sections were dewaxed, rehydrated, and microwaved for 10 minutes in a citric acid-based antigen unmasking solution (Vector Laboratories, Burlingame, CA). Sections were permeabilized with 0.3% Triton X-100 in PBS and blocked with 10% goat serum in PBS. Primary antibodies were incubated overnight at 4°C in PBS, 0.1% Triton, 2% goat serum. Sections were washed twice and incubated with the secondary fluorescent antibodies and DAPI in PBS, 0.1% Triton, 2% goat serum for 1 hour in the dark at room temperature. The sections were washed again and mounted in Mowiol. Stained and fluorescent-labeled sections were analyzed under a Nikon ES800 microscope. Micrographs were taken with a digital spot camera, using equivalent exposure times among sections. For Ntrk3 staining, fresh frozen sections were dried, fixed in methanol at −20°C and washed in PBS, 0.1% Tween 20 before incubation with anti-Ntrk3 antibodies at 1 µg/ml.

For quantitation of immunofluorescent signals, ImageJ 1.42 was utilized. H3K4me3 stained sections were digitally captured and light intensity measured by placing a fixed size circular area over the nuclei of cells and summing all pixels over the given area. At least 6 podocytes and 6 control cells, either mesangial or endothelial, were measured for each of 8 glomerular tufts (at least 48 podocytes and 48 other cells for each genotype). The average signal intensity was then expressed as a ratio of podocyte intensity to non-podocyte cell intensity for each of the glomerular micrographs taken.

For Cre activity detection, the Rosa26-lacZ reporter strain was used [56]. Mice carrying *Cre* ^NPHS2^ and *Paxip1^fl/fl^* were crossed to Rosa26-stop-lacZ:*Paxip1^fl/+^* to generate *Paxip1^fl/fl^*:*Cre* ^NPHS2^:Rosa26-lacZ animals. Kidneys were excised at 1 month of age and stained for β-galactosidase activity as described [57].

### Scanning and Transmission Electron Microscopy

Longitudinal slices of kidneys from PTIP+ and PTIP− mice fixed with 2.5% glutaraldehyde in 0.1M Sorensen's buffer (pH 7.2) for 2 hours at room temperature were processed for scanning electron microscopy following standard procedures. Briefly, after several washes with the Sorensen's buffer alone, the samples were dehydrated by successive washes in graded ethanol solutions, critical point dried, mounted on a stub, sputter coated with gold-palladium, and examined under an AMRAY 1910 field emission scanning electron microscope. Pieces of the kidney cortex (1 mm^3^), fixed with 2.5% glutaraldehyde in Sorenson's buffer for 2 hours at room temperature, were processed for transmission electron microscopy following standard procedures. They were embedded in PolyBed 812 resin (Polysciences Inc.), cut into 1-micron slices and stained with toluidine blue. Sample areas were selected based on the presence of glomeruli and cut into ultra-thin sections for analysis under a Philips CM-100 transmission electron microscope. The selected SEM and TEM images are representative of at least 10 different glomeruli per kidney.

### Isolation of Mouse Glomeruli

Glomeruli were isolated from the kidneys of individual mice by sieving as described [58]. Briefly, 1 month-old mice were sacrificed by CO_2_ inhalation and kidneys were removed. After decapsulation, the kidneys were finely minced on ice and passed sequentially through nylon meshes of 90 and 41 microns (Sefar Filtration Inc., Depew, NY). The glomeruli-enriched fraction (GEF) was retained on top of the 41-micron mesh, while kidney tubules were flushed through. RNA was isolated directly from the mesh.

### RNA Extraction and Reverse Transcription

Total RNA was extracted from the GEF of individual 1-month-old mice using the RNeasy Tissue Micro Kit (Qiagen, Valencia, CA) following the manufacturer's instructions. RNA concentration and purity were determined by nanodrop analysis on an Agilent Bioanalyzer 2100 (Agilent Technologies, Santa Clara, CA). Using the Ovation RNA Amplification System V2 (NuGEN Technologies, San Carlos, CA), 500 ng total RNA was reversed transcribed and linearly amplified into single-stranded cDNA, which concentration and purity were determined by nanodrop analysis on an Agilent Bioanalyzer 2100 (Agilent Technologies).

### Microarray and Real-Time qPCR Analyses

Microarray analyses were done by the University of Michigan Comprehensive Cancer Center (UMCCC) Affymetrix and Microarray Core Facility. The FL-Ovation cDNA Biotin Module V2 kit (NuGEN Technologies, San Carlos, CA) was used to produce biotin-labeled cRNA, which was then fragmented and hybridized to a Mouse 430 2.0 Affymetrix GeneChip 3′ expression array (Affymetrix, Santa Clara, CA). Array hybridization, washes, staining, and scanning procedures were carried out according to standard Affymetrix protocols. Expression data were normalized by the robust multiarray average (RMA) method and fitted to weighted linear models in R, using the affy and limma packages of Bioconductor, respectively [59], [60]. Only probe sets with a variance over all samples superior to 0.1, a p-value inferior or equal to 0.05 after adjustment for multiplicity using the false discovery rate [61], and a minimum 2-fold difference in expression were selected for the analysis. The complete data set is available from the Gene Expression Omnibus database (accession number GSE17709).

Microarray data were confirmed by real-time quantitative PCR analysis. 25–50 ng single-stranded cDNA was amplified in triplicate in a 384-well plate, using the 7900HT Fast Real Time PCR system (Applied Biosystems, Foster City, CA) and expression levels of selected genes was determined by SYBR Green or TaqMan assays (Applied Biosystems). PCR primers pairs and TaqMan probes used in this study are presented in Table S2.

### Chromatin Immunoprecipitation

Glomeruli were isolated from 6 PTIP+ and 6 PTIP− kidneys by sieving as described above. Glomeruli were resuspended in 1 ml PBS and cross linked with 1% formaldehyde for 10 minutes with rocking at room temperature. Chromatin preparation, immunoprecipitation, and PCR analysis was essentially as described previously [13]. Primers pairs for the Ntrk3 locus were as follows: P1, 5′- CAATGTATTTTGCTTCCTTGCC, 5′- AAGAAAGGGTTAGGGGAATCCG *; P2, 5′- AACCCGTGCGTTTCGTAAGG, 5′- GGAGGAAGGAGGAGAAGGAAGATG; P3, 5′- GCATCTTCCTTCTCCTCCTTCCTC, 5′- AAGTCACCAAGTCCCACCTCCTAG; P4, 5′- TTTGCCTTCCCACCGTCTGTTG, 5′- TGCCTTTGAAACGCCGAAC.*

## Supporting Information

Table S1 Podocyte-specific genes that are unchanged after PTIP deletion.
(0.03 MB DOC)

Table S2 Quantitative RT-PCR primer sets and probes.
(0.02 MB DOC)
