# Supplementary material for: Altering a Histone H3K4 Methylation Pathway in Glomerular Podocytes Promotes a Chronic Disease Phenotype
Source: PLoS Genet. 2010 Oct 28;6(10):e1001142. doi: 10.1371/journal.pgen.1001142 (PMC2965754; doi:10.1371/journal.pgen.1001142)
Supplement: Table S1 — Podocyte-specific genes that are unchanged after PTIP deletion. (0.03 MB DOC) [file pgen.1001142.s001.doc]

| **ProbeID** | **Symbol** | **Description** | **UniGene** | **P_value** | **Fold_Change** |
| --- | --- | --- | --- | --- | --- |
| 54631 | Nphs1 | nephrosis 1 homolog, nephrin (human) | Mm.437830 | 0.0208 | 0.3360 |
| 170484 | Nphs2 | nephrosis 2 homolog, podocin (human) | Mm.289099 | 0.4335 | -0.2163 |
| 104027 | Synpo | synaptopodin | Mm.252321 | 0.6247 | -0.0544 |
| 19277 | Ptpro | protein tyrosine phosphatase, receptor type, O | Mm.186361 | 0.0208 | -0.2712 |
| 60595 | Actn4 | actinin alpha 4 | Mm.276042 | 0.6792 | 0.0398 |
| 14107 | Fat1 | FAT tumor suppressor homolog 1 (Drosophila) | Mm.27365 | 0.6247 | -0.0761 |
| 22431 | Wt1 | Wilms tumor homolog | Mm.389339 | 0.7679 | 0.0257 |
| 16917 | Lmx1b | LIM homeobox transcription factor 1 beta | Mm.57111 | 0.7912 | -0.0328 |
| 21412 | Tcf21 | transcription factor 21 | Mm.16497 | 0.0701 | 0.2699 |
| 12577 | Cdkn1c | cyclin-dependent kinase inhibitor 1C (P57) | Mm.168789 | 0.6792 | -0.0494 |
