# Supplementary material for: Altering a Histone H3K4 Methylation Pathway in Glomerular Podocytes Promotes a Chronic Disease Phenotype
Source: PLoS Genet. 2010 Oct 28;6(10):e1001142. doi: 10.1371/journal.pgen.1001142 (PMC2965754; doi:10.1371/journal.pgen.1001142)
Supplement: Table S2 — Quantitative RT-PCR primer sets and probes. (0.02 MB DOC) [file pgen.1001142.s002.doc]

|  | **Forward (5’-3’)** | **Reverse (5’-3’)** |
| --- | --- | --- |
| **Actb** | AGAGGGAAATCGTGCGTGAC | CAATAGTGATGACCTGGCCGT |
| **Nephrin** | ACCCTCCAGTTAACTTGTCTTTGG | ATGCAGCGGAGCCTTTGA |
| **Ntrk3** | TACACGGGACTCCAGAAGCTGACC | CGAAGGCTCAGCGTCTGGAAGA |
| **Padi4** | CTGGCAGAGGGTGACATCATCGAC | TTGGGGATGCCCAGGTACTTGC |
| **Podocin** | GGGACATCTGCTTCCTGGAA | TGATAGGTGTCCAGACAGGGTAAAA |
| **Synpo** | TTCCTTGCCCTCACTGTTCTG | TCCTAGCAGCAATCCACATCTG |
| **Wif1** | CCACCTGAGGAGAGCTTGTACC | TGGCATTCTTTGTTGGGCTTTCC |
| **Wt-1** | GCTCCTGAGGACGCCCTACAGC | TCATACCCTGTGCCGTGGTTGC |
| **S26** | AGTTTGTCATTCGGAACATT | GATCGATTCCTAACAACCTTG |
| **Taqman Probe Assay** | | |
|  | **Assay name (Applied Biosystems)** | **Dye/Quencher** |
| **Prm1** | Mm00726976_s1 (inventoried) | FAM/MGB |
| **Actb** | Endogenous control, mouse actin beta | FAM/MGB |
